# A computational toxicogenomics approach identifies a list of highly hepatotoxic compounds from a large microarray database

**DOI:** 10.1371/journal.pone.0176284

**Published:** 2017-04-27

**Authors:** Héctor A. Rueda-Zárate, Iván Imaz-Rosshandler, Roberto A. Cárdenas-Ovando, Juan E. Castillo-Fernández, Julieta Noguez-Monroy, Claudia Rangel-Escareño

**Affiliations:** 1 School of Engineering and Sciences, Tecnológico de Monterrey Mexico City, Mexico City, México; 2 Computational Genomics Lab., Instituto Nacional de Medicina Genómica, Mexico City, México; Florida International University, UNITED STATES

## Abstract

The liver and the kidney are the most common targets of chemical toxicity, due to their major metabolic and excretory functions. However, since the liver is directly involved in biotransformation, compounds in many currently and normally used drugs could affect it adversely. Most chemical compounds are already labeled according to FDA-approved labels using DILI-concern scale. Drug Induced Liver Injury (DILI) scale refers to an adverse drug reaction. Many compounds do not exhibit hepatotoxicity at early stages of development, so it is important to detect anomalies at gene expression level that could predict adverse reactions in later stages. In this study, a large collection of microarray data is used to investigate gene expression changes associated with hepatotoxicity. Using TG-GATEs a large-scale toxicogenomics database, we present a computational strategy to classify compounds by toxicity levels in human and animal models through patterns of gene expression. We combined machine learning algorithms with time series analysis to identify genes capable of classifying compounds by FDA-approved labeling as DILI-concern toxic. The goal is to define gene expression profiles capable of distinguishing the different subtypes of hepatotoxicity. The study illustrates that expression profiling can be used to classify compounds according to different hepatotoxic levels; to label those that are currently labeled as undertemined; and to determine if at the molecular level, animal models are a good proxy to predict hepatotoxicity in humans.

## Introduction

Toxicogenomics is a field that integrates data from high-throughput technologies into research on conventional toxicology. Major goals include elucidating the molecular mechanisms of toxicity, identifying potential biomarkers for exposure to toxic substances [1] and developing methods to predict toxic effects of compounds used by the pharmaceutical industry [2]. From the economics viewpoint toxicogenomics provides information useful to decide whether to proceed further in the drug development. Nevertheless, the main goal is that of human health. The liver and the kidney are the most common targets of chemical toxicity, due to their major metabolic and excretory functions [3]. However, since the liver is involved in biotransformation [4], many currently and normally used drugs could affect the liver adversely. Drug compounds are absorbed from the gut and directly transported to the liver through the portal vein receiving relatively high concentrations before elsewhere in the system.

Drug-induced liver injury (DILI) refers to an adverse drug reaction, which remains a major problem in drug development and pharmacotherapy representing clinical and financial challenges. Compounds are labeled according to Federal Drug Administration (FDA) approved labels [5] using DILI-concern scale. This scale can be divided in three levels: No DILI-concern when the drug is clean in all three drug labelling sections, Less DILI-concern if it is mentioned in the section of adverse reaction or in the Warnings and Precautions label section, but its severity is low. Less DILI-concern shows elevated levels in classical clinical chemistry markers like aspartate aminotransferase (AST) and alanine aminotransferase (ALT). Most DILI-concern for its potential to cause liver injury in human from cases previously reported. Most DILI-concern drugs lead to severe liver damage, or even death.

Many drugs act by binding to protein targets altering their function, one foundational assumption in toxicogenomics is that exposure to a toxicant leads to altered gene expression either directly or indirectly ([6], [7], [8], [9], [10], [11]). Altered proteins in the liver can have functional and behavioral effects on other protein coding genes that may result in liver damage. Therefore, molecular biology techniques involving gene profiling have been developed to establish correlations between gene expression and drug toxicity on both in vitro and animal models. In the clinical setting, a number of genes or proteins have already been identified as potential predictive markers of drug activity and their use could be progressively implemented for drug safety. Important initiatives, such as The Japanese Toxicogenomics Project (TGP), developed by the Japanese National Institute of Health Science, the National Institute Biomedical Innovation and 15 pharmaceutical companies [12] have been launched. TGP is a five-year project completed in 2007 that has established a large-scale toxicogenomics database known as Genomics-Assisted Toxicity Evaluation System (TG-GATEs) [13], [14]. TG-GATEs is a large-scale database of transcriptomics and pathology data potentially useful for predicting the toxicity of current and new chemical compounds. Transcriptomics data was generated using Affymetrix GeneChip arrays human HGU133plus2 and rat RAE230 2.0. Approximately 170 chemical compounds, primarily medicinal, were tested at multiple doses. Gene expression was measured in three biological models: rat and human hepatocytes treated with the chemicals in vitro and conventional in vivo toxicology studies for single dose and repeated dosing on rats. The in vivo set of studies explored gene expression in liver and kidney although mainly in liver. In vitro studies include duplicate samples collected at four doses Control, Low, Middle, High across three time points 2hr, 8hr, 24hr of exposure. Single dose in vivo studies include triplicate samples collected at the four doses across four time points 3hr, 6hr, 9hr, 24hr. The repeat dose in vivo studies collected samples at 4d, 8d, 15d, and 29d following daily dosing with the same four doses (d = days).

Analysis of such large and comprehensive toxicogenomics database has been a great challenge ever since it was released through the Critical Assessment of Massive Data Analysis (CAMDA) [http://dokuwiki.bioinf.jku.at/doku.php] challenge in 2013 [15]. Even though it includes a large collection of samples, any classical statistical analysis will be based on how many observations of the same variable we have access to. In this case, only two for the in vitro models and three for the in vivo rat model. As for the number of variables, this is given by the total number of different sources of RNA samples which includes up to 170 chemicals at 4 doses across 3 or 4 time points depending on the model reaching thousands of variables. Hence, performing a global analysis of this dataset remains an open problem. Previous work attempting to use the full data set includes the identification of the main biological processes for each compound [16], but it is done on dose by dose comparison, it does not involve the full set of variables simultaneously as we do with the time course approach. Another approach identified four genes in Human in vitro samples that may predict early response to cytotoxicity and were later validated in Rat in vitro and in vivo samples [17]. The diversity of the TG-GATEs data structure is suitable for different approaches to a given problem whether is feature selection, class discovery or prediction analysis. At the same time, a variety of specific problems could be targeted, for instance, identification of synergistic and antagonistic compounds through gene profiling. In this case, hypothesis driven strategies would retrieve a specific subset of samples for the analysis. On the other hand, a data driven strategy, would attempt to use large collection of samples and search for patterns that will in turn, generate hypotheses for further evaluation and validation. In this work we used both approaches combined in order to classify compounds by their hepatotoxicity level, matching DILI-concern FDA labelling for those available and attempting to label others for which no DILI-concern label has been declared. We developed a new computational strategy that applies machine learning techniques to summary statistics of ranked genes that allows to mine chemical compounds using all information about doses and time simultaneously.

## Materials and methods

### Data

From the complete list of 170 compounds from TG-GATEs, only 48 were administered to in vivo and in vitro samples in rat and human models. A subset of 4,578 microarrays interrogating those 48 compounds, mostly drugs were used to analyze hepatotoxicity patterns through gene expression profiling. Only three models were included in the analysis: Rat in vivo (2,280 microarrays), Rat in vitro (1,146 microarrays) and Human in vitro using primary hepatocytes (1,152 microarrays). For each individual compound there was a control and three administered dose levels {Low, Middle, High} during a 24-hour period. Three time measurements were sampled for in vitro models and four for the in vivo model on gene expression microarrays including two and three biological replicates respectively. Raw data in the form of.CEL files, each of which corresponding to a microarray were stored for later access from R through a SQL database. Human primary hepatocytes were processed using Affymetrix HGU133Plus2, and animal samples on the GeneChip Rat Genome 230 2.0 which is known to be a powerful tool for toxicology (Affymetrix, Santa Clara,CA, USA).

#### Data pre-processing

Data storage, access and manipulation was done using a relational database. Raw-data analysis included quality metrics using R and bioconductor libraries affy and oligo. Data were normalized using Robust Multiarray Average algorithm (RMA) [18] with no background correction due to the bimodal effect after correction and Quantile normalization [19], [20].

### Strategy for analysis

We propose a strategy that combines data from the three models (Human in vitro, Rat in vitro and Rat in vivo) with dose levels (Ctrl, Low, Mid, High) in a time series approach on each of the 48 selected compounds. Two main goals motivate the analysis: To determine if at the molecular level, animal models are a good proxy to predict hepatotoxicity in humans and to classify compounds by toxicity levels in human and animal models through patterns of gene expression. To achieve these goals, we combined machine learning algorithms with time series analysis to classify genes whose absolute or relative expression varies over time, incorporating at the same time the correlation structure across time points. The time series method proposed by Tai and Speed [21], which is specifically designed for microarray data where moderation and replication are used was implemented using the timecourse package https://www.bioconductor.org/packages/release/bioc/html/timecourse.html.

### Statistical approach

#### Time series analysis

The dynamic nature of the data was explored using a time series analysis on every compound with the timecourse package [22] available from Bioconductor. For every selected subset, genes were classified according to a multivariate empirical Bayes (MB) statistic [23] for replicated microarray time course data. Genes were ranked based on large absolute or relative amounts of change over time as a function of the drug concentration in relation to their replicate variances. The summary statistic for the ranking was the MB statistic which is a one-sample multivariate empirical Bayes to select differentially expressed genes from replicated microarray time course experiments.

#### Machine learning

Further analysis using machine learning methods allowed us to learn interesting patterns on already ranked genes through unsupervised hierarchical clustering on MB values after filtering them with Median Absolute Deviation (MAD) [24] to contrast relevant from irrelevant compounds. Unsupervised hierarchical clustering was applied to MB values to identify clusters of genes with similar importance across compounds. Importance was defined based on the ranking determined by the time course analysis. Pathway enrichment analysis was conducted using Gene Set Enrichment Analysis (GSEA) [25], which ranks genes based on the correlation between their expression and the class distinction or phenotype, the “pre-ranked GSEA” works with any summary statistic as ranking metric, which in our case was the MB statistic.

## Results

Database implementation and data retrieval through R made all of the time course analyses time-efficient. The list of the 48 chemical compounds used in this work is presented in Table 1.

**Table 1:** List of 48 compounds found in common for Human in vitro, Rat in vitro and Rat in vivo. All previously reported as highly toxic.

Abbreviation	Compound Name	Abbreviation	Compound Name
AA	Allyl Alcohol	GFZ	Gemfibrozil
ADP	Adapin	HCB	Hexachlorobenzene
AM	Amiodarone	HPL	Haloperidol
ANIT	Naphthyl Isothiocyanate	IM	Indomethacin
APAP	Acetaminophen	INAH	Isoniazid
APL	Allopurinol	KC	Ketoconazole
ASA	Aspirin	LBT	Labetalol
AZP	Azathioprine	LS	Lomustine
BBr	Benzbromarone	MP	Methapyrilene
BBZ	Bromobenzene	MTS	Methyltestosterone
CBZ	Carbamazepine	NFT	Nitrofurantoin
CCL4	Carbon Tetrachloride	OPZ	Omeprazole
CFB	Clofibrate	PB	Phenobarbital
CIM	Cimetidine	PH	Perhexiline
CMA	Coumarin	PhB	Phenylbutazone
CPA	Cyclophosphamide	PHE	Phenytoin
CPZ	Chlorpromazine	PTU	Propylthiouracil
DFNa	Diclofenac	RIF	Rifampicin
DZP	Diazepam	SS	Sulfasalazine
ET	Ethionine	TAA	Thioacetamide
FP	Fluphenazine	TC	Tetracycline
FT	Flutamide	TRZ	Thioridazine
GBC	Glibenclamide	VPA	Valproic Acid
GF	Griseofulvin	WY	Wy-14643

**Fig 1:**
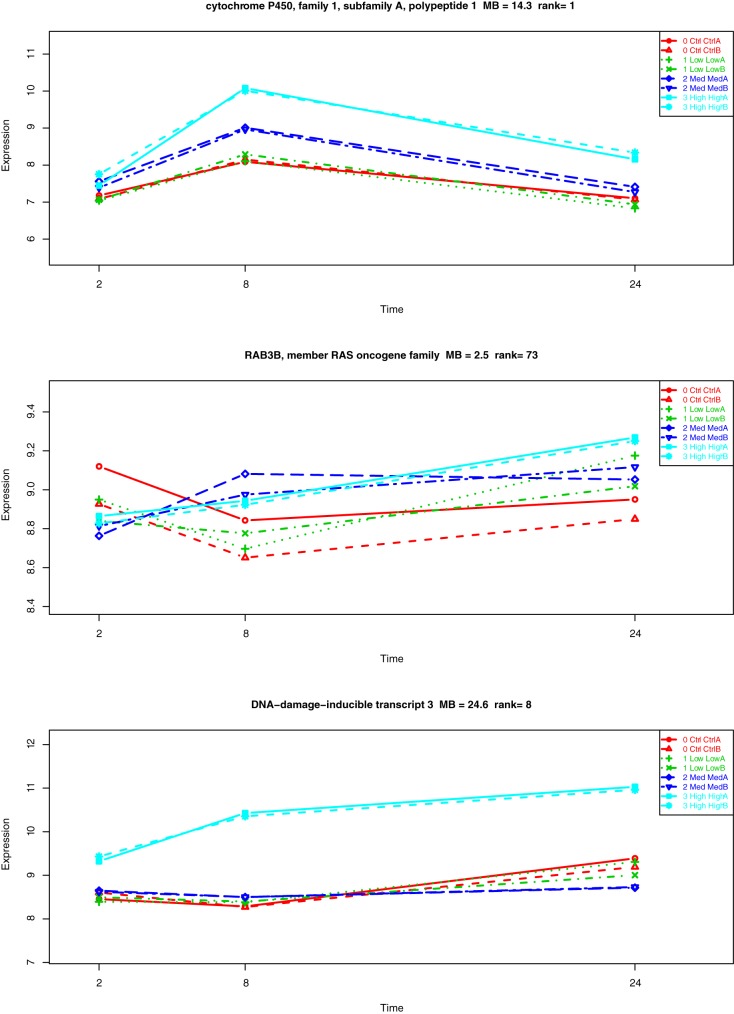
Time course approach on different compounds. (a) Carbon Tetrachloride on Human in vitro samples (b) Aspirin on Human in vitro samples and (c) Phenytoin on Rat in vitro samples. Colors are assigned by dose: Control(red), Low (green), Middle (blue), High (cyan). On the x-axis the time measurements 2hr, 8hr, 24hr; on the y-axis the gene expression values at each time point.

Genes were ranked based on variation over time as a function of the drug concentration in relation to their replicate variances. In other words, time series analysis considered three parameters to determine the gene ranking. The first parameter is about differential expression across time, there should be substantial changes in expression values between two or more consecutive time points. This is exemplified by the dynamic range of the y-axis on plots in Fig 1, in (a) gene is ranked #1, and (b) gene is ranked #73. The second parameter shows statistical robustness considering the case of low replication. Here, replicates indicated by same color should almost lie one on top of the other indicating a strong similarity as we observe in Fig 1 plots (a) and (c) ranked #1 and #8 respectively, but definitely not in plot (b) with much lower ranking of 73. The third parameter is about differential expression between conditions, doses in our case. We should expect a clear separation between two or more conditions as it is the case for plot in Fig 1 (a) where we see that even though control and low doses are almost identical, a separation between them and middle and high doses is clear. The multivariate Bayes or MB statistic summarizes information from these three parameters into a value which is also translated into a rank.

Carbon Tetrachloride (CCL4) is a solvent for manufacturing organic compounds, its primary effects in humans are on the liver, kidneys, and central nervous system. Poisoning by inhalation, ingestion or skin absorption is possible and may be fatal. Administration of this compound in human hepatocytes showed *cytochrome P450 family 1 subfamily A*, at the top of the list ranked #1. This gene is part of a pathway for xenobiotic metabolism. In Fig 1 a) we observe clear differential expression at time 8 suggesting that the higher the dose the larger the impact of *CCL4* on this gene. Aspirin (ASA) time course in contrast, does not show any of the patterns observed in (a), replicates have an erratic behavior and differential expression represented by the values on the y-axis are almost negligible and unable to separate effects by doses, therefore ranked #73. Phenytoin is an anticonvulsant used to treat a wide variety of seizures. Reports about DNA damage have been published [26]. Plot c) shows how *DNA damage inducible transcript 3* is ranked #8 with MB = 24.6 when high dose is administered. It is the kind of information we would look for in an attempt to identify hepatotoxic biomarkers either by time of exposure and/or dose concentration.

### Unsupervised hierarchical clustering analysis

**Fig 2:**
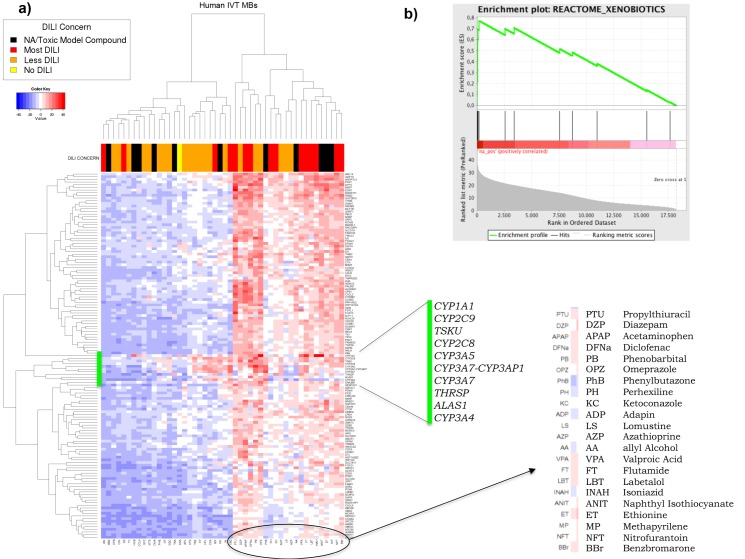
Hierarchical clustering of top ranked genes in Human in vitro model. In the x-axis the compounds are shown and in the y-axis the selected genes by MB and MAD. In red, values with high MB statistic and in blue negative MB values. Colored bar on the top shows DILI concern, black means unassigned or model compound, yellow indicates No DILI, orange Less DILI and red Most DILI. Gene set enrichment analysis (top-right) was done with pre-ranked GSEA. Marked with a green vertical bar are genes that remain significant across the majority of compounds and the list on the far right shows the list of compounds with the highest statistical significance.

Once ranked, a list of 160 most variable ranked genes according to MAD of the Human in vitro samples were selected for hierarchical clustering analysis on the MB values (List available in supplementary files S4 Table). Clusters of highly significant genes according to the MB statistic were identified, see Fig 2.

**Fig 3:**
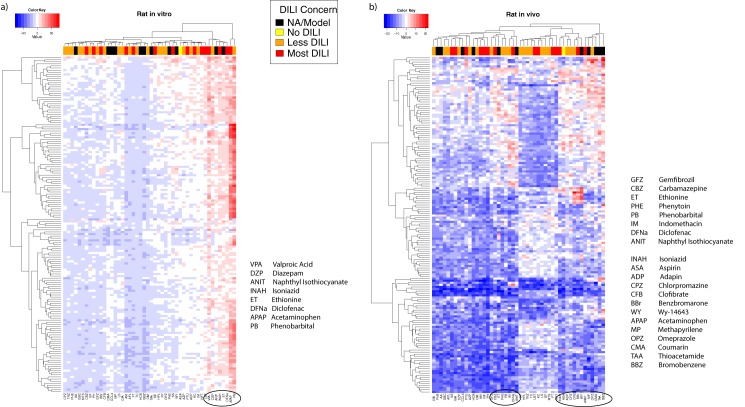
Hierarchical clustering of top ranked genes in both of the Rat models in vivo, in vitro. In red values with high MB statistic and in blue negative MB values. Rat in vitro (left) and Rat in vivo (right). Colored bar on the top shows DILI concern, black means unassigned or model compound, yellow indicates No DILI, orange Less DILI and red Most DILI.

A similar analysis on the two Rat models was performed showing a rather different pattern but interestingly enough, a very similar cluster of compounds to that in the Human in vitro model. Fig 3, shows the resulting heat maps. Image on the left is for Rat in vitro model with 160 genes after MAD and Rat in vivo model is shown on the right with 160 genes after MAD. Both generate different dynamic ranges for the MB-statistic values shown by the blue to red intensity spectrum. The Rat in vitro model correlates better with compounds labeled as “Most DILI-concern” shown in red in top bar. There was however, six compounds that despite the clear difference in the heat map patterns, appeared as highly significant in Human in vitro, Rat in vitro and even Rat in vivo consistently: ANIT, APAP, DFNa, ET, INAH, PB, VPA.

## Discussion

A paradigm shift in toxicology to explore the possibility of replacing the animal model with in vitro assay supported with toxicogenomics research motivated the development of the TGP. The TG-GATEs database contains both in vitro and animal data to be able to address this question. Patterns of genes (rows) and compounds (columns) vary clearly according to significance levels based on the MB values for each model. When we compare left and right plots in Fig 3 the blue and red intensities are given by the dynamic range of MB values shown in Table 2. Rat in vitro reached high significance levels, up to 78.37 whilst Rat in vivo could only reach 28.84 possible indicator of better sensitivity or better experimental control for in vitro studies or both. Aside of MB values, the lists of top ranked genes in both Rat models did not show a good level of consistency or reproducibility. We could argue that in vitro assays may not be a good replacement for animal model in rats. However, the doses and the times were not exactly the same, they were adjusted for in vivo studies. This may suggest that more work needs to be done adjusting doses and exposure time in animals since it was reported missing data due to animals died before the completion of the time interval for sampling.

**Table 2:** Dynamic range of MB statistics.

	Human in vitro	Rat in vitro	Rat in vivo
min	-25.65905	-25.20966	-26.76873
max	42.42583	78.36532	28.83527
No. genes	160	1000	160

For our second goal, classifying compounds by toxicity levels in human and animal models through patterns of gene expression shown in Fig 2 and Fig 3. Even though patterns of genes are not as consistent as one would expect, when we look at the list of compounds classified by those top ranked genes, a subset of 6 appear consistently in the three models shown in Table 3. The fact that 5 out of 6 are known to be highly toxic marked in red on the DILI-concern bar at the top of the heat map and that they cluster together with other toxic model compounds marked in black suggest that those labeled as Less DILI-concerned (marked in orange) should be updated to Most DILI-concern according to FDA labelling [17] and administration of those must be closely observed.

**Table 3:** All previously reported as highly toxic compounds found in common for Human in vitro, Rat in vitro and Rat in vivo models.

Abbreviation	Compound	Hu in vitro	Rat in vitro	Rat in vivo
VPA	Valproic Acid	√	√	–
INAH	Isoniazid	√	√	√
ET	Ethionine	√	√	√
APAP	Acetaminophen	√	√	√
PB	Phenobarbital	√	√	√
ANIT	Naphthyl Isothiocyanate	√	√	√
DFNa	Diclofenac	√	√	√

## Conclusions

Despite the large number of microarray data available from the TG-GATEs, its complex structure with many groups and lack of replication makes it difficult for pattern recognition approaches. Feature selection, clustering and other classification techniques did not perform well directly on gene expression values. Combining information from dose, time, animal or human models on each compound requires a more comprehensive method. The analysis by time series developed by Tai and Speed provided a methodology to rank genes using all their features such as dose, time of exposure and differential expression even with low replication. Classification by ranking using the MB statistic, a one-sample multivariate empirical Bayes that selects differentially expressed genes from replicated microarray time course experiments, allowed us to summarize time changes, dose concentration, quality of replicates and significant differential expression representing a fast and appropriate way to reduce complexity of the highly diverse data structure. Unsupervised hierarchical clustering was later applied to MB statistical values showing rather different patterns for Human in vitro, Rat in vitro and Rat in vivo models indicating that animal models can indeed correlate with human model for highly toxic compounds but shows a diverse pattern in general.

Unsupervised machine learning techniques provided a set of genes capable of classifying compounds as DILI-concern toxic according to the FDA-approved labelling. Compounds already known to be highly toxic clustered with poisonous model compounds suggesting a possible list of toxic biomarkers. The case of Phenobarbital (PB) might be of interest, it is labeled as Less-DILI-concern but appeared clustered together with highly toxic compounds. The analysis pipeline used for this work can be reproduced on other data sets involving a different list of compounds and all doses or just a particular selection of doses.

## Supporting information

S1 Table MB values for the Human in vitro model. This is the matrix that contains the MB values for the Human in vitro model obtained from the Timecourse package.
(CSV)

S2 Table MB values for the Rat in vitro model. This is the matrix that contains the MB values for the Human in vitro model obtained from the Timecourse package.
(CSV)

S3 Table MB values for the Rat in vivo model. This is the matrix that contains the MB values for the Human in vitro model obtained from the Timecourse package.
(CSV)

S4 Table Top 160 genes for each experiment model. Table that includes the top 160 genes of each experiment model ordered by MAD using the MB statistic as input. In the second tab is the order of the compounds presented in each of the hierarchical clustering dendrograms.
(XLSX)
